# Differences in the Perception of Social Support Among Rural Area Seniors—A Cross-Sectional Survey of Polish Population

**DOI:** 10.3390/ijerph15061288

**Published:** 2018-06-19

**Authors:** Paweł Chruściel, Teresa Kulik, Klaudia Jakubowska, Dorota Nalepa

**Affiliations:** 1 Department of Basic Nursing and Medical Teaching, Chair of Development in Nursing, Faculty of Health Sciences, Medical University of Lublin, 20-081 Lublin, Poland; klau.jakubowska@gmail.com; 2 Chair of Public Health, Faculty of Health Sciences, Medical University of Lublin, 20-093 Lublin, Poland; teresa.kulik@gmail.com; 3 Faculty of Social Sciences and Humanities, State Higher Vocational School Memorial of Prof. Stanisław Tarnowski in Tarnobrzeg, 39-400 Tarnobrzeg, Poland; nalepad1@wp.pl

**Keywords:** social support, social networks, elderly, long-term care, institutional care

## Abstract

*Introduction and objective*: Social support constitutes an important determinant of an elderly person’s health and of functioning in his or her living environment. It depends on available support networks and the type of help received. Measurement of social support should encompass both its structure and the functions it fulfills, which enables detailed assessment of the phenomenon. The aim of the study was to compare the perception of social support among rural area seniors provided with institutional care with those living in a home setting. *Material and method*: Using the diagnostic survey method and the technique of the distribution of a direct questionnaire, 364 respondents from rural areas were examined: those living in an institutional environment (*n* = 190) and those living in their home (natural) environment (*n* = 174). The respondents were selected on the basis of a combined sampling method: proportionate, stratified, and systematic. Variables were measured with the following questionnaires: Courage Social Network Index (CSNI) and Social Support Scale (SSS). *Results*: The living environment has been proved to differentiate average values of support both in the structural and functional dimensions in a statistically significant way (*p* < 0.001). An untypical phenomenon was higher average values pertaining to emotional bonds, frequency of direct contacts, and help received in the group of respondents living in an institutional environment. *Conclusions*: The living environment and demographic variables affect the perception of social support among elderly people. Full-time institutional care of a senior citizen leads to the deterioration of social support; therefore, keeping an elderly person in a home environment should be one of the primary goals of the senior policy.

## 1. Introduction

Members of the support network, their willingness, and the type of support provided by them play a crucial role in the successful functioning of an elderly person in the environment. A support network typically encompasses family members and frequently people who are not related but who are important to an individual for various reasons. The most valuable and usually unfailing sources of help for an elderly person are natural sources of support (most frequently, family members and life partner) who, in comparison to institutional sources, have a more beneficial influence on a senior citizen and do not lead to his or her stigmatization [1,2,3]. Considerations on social support seem to be especially significant with reference to the health and social situation of elderly people, because the needs and expectations of this group of society should be regarded as a priority in the context of independent functioning and institutional care. Furthermore, epidemiological and demographic trends and changes in the family structure, as well as a decline or a complete lack of a support network, directly contribute to the increased need for institutional care and thus the deprivation of natural sources of support for seniors [4]. Not only the aforementioned factors but also demographic variables play a significant role in the seniors’ perception of social support. Better assessment of social support has been proved to occur usually in women, younger individuals, those having partners and those who are better-educated [5,6,7,8,9,10].

Undertaking this issue is important for several reasons. One is the progress of demographic changes. Individuals over 60 years of age constitute more than 6.8 mln. (17.8%) of the Polish population. According to prognostic data, in 2050 the number of Polish residents over 65 will increase by 30% (approximately 18 mln. people) while individuals over 80 will constitute 32% of the elderly population (more than 3.5 mln. people). The values are estimated to increase by about 180% in urban areas and around 225% in rural areas [11,12]. Despite the changing structure of households and functions fulfilled by a family, an informal support network is the primary and fundamental source of help both in health and in illness. Moreover, an informal support network most frequently undertakes long-term care of a dependent elderly person in his or her place of residence. It is assumed that the readiness to take direct care of the eldest family members is more typical of rural households where large multi-generational families still predominate, a situation conducive to close relationships. On the other hand, authors emphasize that rural area seniors are characterised by lower social support than those living in urban areas [5,7,9,10]. For this reason, the study is focused on the situation of elderly people living in rural areas. In order to emphasize differences in perception of social support, senior citizens under full-time institutional care were selected as a reference group.

The semantic diversity of social support is not conducive to giving it a precise definition, but it oscillates around supportive actions between a person giving support and the person receiving it [13]. Present-day social support can be described as a kind of a transaction between a giver and a recipient of help, through which values, tools, information, and emotions can be exchanged in connection with the fact that the participants of the exchange belong to a particular support network [14]. In the domestic literature this phenomenon is usually presented one-sidedly—usually in qualitative categories. Nevertheless, the character of social support should be considered comprehensively in the context of two supplementary dimensions: quantitative (structural support) and qualitative (functional support) [11,12,15,16]. Furthermore, structural support should be identified with the existence and availability of particular support networks—people ready to provide help to a person expecting support [14,17,18]. In accordance with this general division, two categories of support sources can be distinguished:

a. informal, constituting the so-called original (natural) network, encompassing family members, friends, neighbours, and co-workers;
b. formal, creating the basis for the secondary network, that is people who provide help professionally and institutions of a supportive and protective character, religious denominations, or associations [19,20,21].

On the other hand, functional support describes the type of relations between a supporting person and a supported one. Several types of functional support can be distinguished (depending on the content of social exchange):

a. informational support, consisting in exchange and provision of information aimed at helping the recipient overcome difficulties through understanding his or her situation and the nature of the problem;
b. instrumental support, that is, providing direct and real help in the form of services and actions (e.g., feeding, supplying medication, providing shelter, purchase of certain products). A popular form of instrumental support is financial help;
c. evaluative support, that is assuring a person that he or she has such resources, capabilities and skills which are important for a given support network and helpful in the successful functioning of its particular members;
d. emotional support, connected with expressing supportive and reassuring emotions, building a sense of care and trust [14,18,19,22,23].

The hypothesis has been verified whether the provision of stationary institutional care for seniors leads to the deterioration of social support.

### Objective of the Study

The aim of the study was to compare the perception of social support among rural area seniors provided with institutional care with those living in a home setting.

## 2. Material and Method

### 2.1. Questionnaires

The present study uses a quantitative strategy in the form of a diagnostic survey of a cross-sectional character. Variables were measured with standardized tools which enable an in-depth analysis of the character of social support:

a. The Courage Social Network Index (CSNI) was used for quantitative evaluation of social support. Its construction was based on the model of the informal networks’ role with reference to the relationships with members of eight different sources of support: partner, parents, children, grandchildren, relatives, co-workers, neighbours and friends. The questionnaire is comprised of five questions referring to each source of support. The result of the scale is described with a general percentage value ranging from 0 to 100%, with a higher percentage signifying a better perception of structural support (SS)—interpretation of the results has a positive direction. Apart from the general result, four dimensions were distinguished within the scale: network structure (NS; question 1—range from 0 to 7), emotional bonds (EB; questions 2 and 3—range from 0 to 3), frequency of direct contact (FC; question 4—range from 0 to 5), and help received (HR; question 5—range from 0 to 5). Psychometric parameters of the scale were found to be satisfactory (Chronbach’s alpha coefficient of reliability ranged from 0.61 to 0.86 depending on the subscale) [24,25]. Despite the fact that the CSNI scale offers an opportunity to calculate only the general result, an analysis of its particular components was carried out. An argument for a more detailed analysis of the variable was the need for extension of the results and familiarisation of the issue.
b. The Social Support Scale (SSS) was used for qualitative evaluation of social support. The structure of the scale was based on the concept of social support which assumes that there are certain kinds of support derived from specific groups (sources): family, friends, neighbours, co-workers, formal and informal organizations, professionals, and service centers. The questionnaire, containing 24 items, provides information on functional support (FS) received by the respondent, and its four types: informational (INF), instrumental (INS), evaluative (EVA), and emotional (EMO). The outcome of the general level of functional support ranges between 24 and 120 points, and each of the support types between 6 and 30 points. Interpretation of the outcomes has a negative direction—a lower number of points is associated with higher functional support and its types. Reliability of the tool was assessed as satisfactory (Spearman-Brown’s coefficient > 0.70) [22].

The Hodgkinson’s Abbreviated Mental Test Score (AMTS) was used to assess the cognitive functions of the respondents. The scores of this test were not statistically analysed because the questionnaire was utilized solely to check cognitive functions of potential respondents to qualify them for the study. Seniors scoring at least 6 out of 10 points were included in the study. Severely or mildly disordered cognitive functions were exclusion criteria for nearly half of potential respondents in both of the groups. The rest of the seniors (51%) showed appropriate cognitive function.

Additionally, a short questionnaire compiled by the authors was applied to determine demographic data (sex, age, education level, and marital status). The survey questionnaires were designed to be completed independently by the respondents. The research was fully approved by the local Bioethical Commission of the Medical University of Lublin (KE-0254/86/2015).

### 2.2. Respondents

A representative group of 364 respondents from rural areas qualified for the research. The respondents were selected on the basis of a combined sampling method: proportionate, stratified without replacement, and systematic. The first type of sampling served to select—in quantitative and qualitative terms—the care-giving institutions and districts of the Lublin Region (Poland). On the other hand, the systematic scheme enabled reaching particular respondents, in accordance with the assumed distance between individuals (*k* = 37). The sampling frame consisted of confirmed official data. The authors decided to choose the Lublin Region to represent the population of Polish seniors due to its agricultural character and considerable percentage of senior residents. Furthermore, the Lublin Region is one of those characterized by the highest degree of institutionalized care for seniors. Having fulfilled the specific qualification criteria (age above 60 and mental condition enabling completion of the questionnaires—a positive score of the AMTS), each respondent confirmed in writing his or her willingness to take part in the study. The respondents were divided into two groups:

a. group A—those living in full-time institutional care centers who represent the institutional environment (*n* = 190);
b. group B—those living in natural conditions who represent the home environment as a reference group (*n* = 174). Introducing this reference group into the study allowed an exposition of possible differences in the perception of social support.

Demographic diversity of the respondents is presented in Table 1.

### 2.3. Statistical Analyses

The analysed variables had a quantitative character. Basic descriptive statistics were calculated for them, with the Kolmogorov-Smirnov test which demonstrated that the distribution of the majority of the variables was not consistent with the normal distribution. Nevertheless, all variables fulfilled an assumption about not exceeding the skewness value of <−2; 2>, so a decision was taken to carry out parametric analyses—Student’s *t*-test for two independent groups. In the case of demographic variables such as education and marital status, the application of the nonparametric Kruskall-Wallis test was necessary because of considerable differences in the number of respondents in the groups. Moreover, the Kuskall-Wallis test results were checked by the Dunn post hoc explanatory test and Bonferroni correction. The statistical analyses were performed with IBM SPSS Statistics 23 package. The test results at the level of *p* ≤ 0.05 were considered statistically significant.

## 3. Results

The analysis of structural support referred to the following elements: network structure, emotional bonds, frequency of direct contact and help received. On the other hand, functional support was studied in the context of its four types: informational, instrumental, evaluative, and emotional. Moreover, a general result was calculated within each of the examined support types. It was evidenced that the living environment influenced average values of support both in the structural and functional dimensions in a statistically significant way (*p* < 0.001, Table 2 and Table 3). As regards structural support and its elements, the respondents living in the institutional environment differed statistically from the respondents living in the home environment, and differences in this area were strong (*d* > 0.8). The respondents living in their natural environment evaluated structural support better and had a more developed network structure (higher average values). There is a significant disproportion of average values for structural support between the two groups studied (M_A_ = 38.22 vs. M_B_ = 66.13). An untypical and at the same time interesting phenomenon is higher average values with respect to emotional bonds, frequency of direct contact, and help received in the group of respondents living in the institutional environment (Table 2).

An attempt was made at explaining differences observed in the respondents from both environments. Thus, the attention was focused on demographic variables to carry out appropriate tests (Table 4 and Table 5).

Females were revealed to assess structural support in a better way and have a more extended structure of networks than males. Similar differences were noticed in the case of emotional bonds, frequency of direct contacts, and readiness to be given help. These differences produced a medium-sized effect (0.3 < *d* > 0.5).

The older respondents gave structural support and readiness to get help a higher assessment, though the differences were not statistically significant (*p* ≥ 0.05). The younger ones rated the remaining elements higher, but only that of stronger emotional bonds was statistically significant (*p* < 0.001). That means the younger respondents had closer emotional bonds within their support networks. Both education and marital status significantly differentiated structural support and all its elements (*p* < 0.05) with quite low intensity (0 < η^2^ > 1). The analysis of intergroup differences made it possible to conclude that the respondents with higher education assessed structural support, structure of their network support, emotional bonds, frequency of direct contacts, and readiness to get help, as lowest.

The widowed and divorced respondents rated structural support better than those who were married. The same group of respondents had a more extended structure of network support than the single and married seniors and the differences were of considerable intensity (η^2^ = 0.72). The divorced respondents were characterised by closer emotional bonds with members of their network support. On the other hand, the widowed ones more frequently experienced direct contacts with individuals who were important to them and they could receive help from them easily in comparison with the remaining groups of respondents.

The respondents living in the home environment are characterized by lower average values of functional, instrumental, evaluative, and emotional support, which indicates that it is they who more highly evaluate the aspects of support under discussion (*p* < 0.05). The intensity of difference in average values between the studied groups is moderate (0.3 < *d* < 0.5). Informational support, despite being different between the examined groups, does not present a statistical interpretation value (*p* > 0.05).

Comparing males and females, the latter assessed functional support better along with all its types. However, no significant differences were noticed in the informational and instrumental scope. Respondents’ age and education did not differentiate the perception of functional support (*p* ≥ 0.05). All the significant differences were characterised by low intensity (0 < *d* < 0.3). For the single respondents, the perception of emotional support was at a higher level than for the widowed ones and the significance of the difference was slight (0 < η^2^ < 1).

## 4. Discussion

The research results suggest that separating seniors from natural sources of support leads to the deterioration of social support. The provision of institutional care for seniors is associated with a change of perception of social support, particularly with reference to functional dimension and then to its structure. The aforementioned findings are confirmed in other authors’ research on seniors’ social support.

### 4.1. Functional Support

It was found that the results of the authors’ own research were consistent with the results achieved by Kurowska and Błaszczuk [5]. These authors conducted a survey whose participants were elderly people under institutional care. The survey provided information on the average level of functional support. With respect to particular types of support, gradation was the following: emotional support was evaluated the highest, followed by informational and instrumental support, while evaluative support was regarded as the worst. The respondents evaluated as highest this type of support which is considered scarce but extremely significant from the perspective of elderly people. Emotional support is particularly important to those senior citizens who have been cut off from their natural sources of support due to various reasons, because this type of support enhances their self-esteem and improves their wellbeing. An increased amount of emotional support could originate from the secondary source of support, that is from the personnel of an institution. Moreover, in comparison with other types of support, emotional support is the most popular and the least demanding of its provider. Emotional support is deemed to have a beneficial effect on the mental condition and quality of life of the person experiencing it [5,21,22].

On the other hand, the type of support estimated at the lowest level, that is evaluative support, is identified with assuring a person of his or her importance to a given support network. In the case of the respondents participating in the authors’ own research, such an assessment may be due to the change from the home environment to an institutional one, which results in a deteriorated support network. Change in the place of residence is connected with leaving the former support network and thus with ceasing to be a valuable member of it. A different interpretation of the lowest assessment of evaluative support is suggested by Kurowska and Błaszczuk [5]. According to their interpretation, evaluative support is not noticed by the respondents. However, in the opinion of the authors of the present study, the conviction that this kind of support is not perceived by the respondents is not true, and its low evaluation can result from lack of access to it in a situation of being under full-time institutional care. The research of Glińska and colleagues [26] concerning social support confirms an increased need for emotional support. Just under 1/3 of the respondents considered this type of support to be commensurate with their current needs. The nursing personnel was regarded as the primary source of this type of support, because support from family members was not possible.

Low evaluation of instrumental support among the respondents from both of the examined environments is not surprising. Taking into account seniors’ income, namely, old age pensions and disability pensions, they usually have to cope with inadequate financial means. Moreover, seniors’ low budgets are frequently a cause for financial support being provided by their family members. On the other hand, an elderly person who can rely on help from informal caregivers (children, grandchildren) in everyday activities such as buying food and medication or paying for their transport, could evaluate this kind of support lower, which is justifiable because he or she does not have to perform these actions on his or her own.

### 4.2. Structural Support

Research for the present study showed that respondents representing the natural living environment evaluated social support at a high level (M_A_ = 38.22 vs. M_B_ = 66.13). The fact that the senior citizens living in their own homes, especially with children and grandchildren, evaluated social support higher is connected with a strong sense of belonging and facilitates cherishing of the relationships. A high level of structural support among these respondents indicated a much more developed support network. The satisfactory results with respect to the support received are confirmed by research carried out by Wróblewska and Iwaneczko [27] in which the respondents were satisfied with the support and interest expressed by institutional caregivers and family. In the research of Jaracz and colleagues [28] and Drageset and colleagues [29] the respondents were characterized by a lower level of social contact. The results obtained by the authors of the present study partially confirm the above-mentioned theses—functional support and its types were assessed lower by the respondents from the institutional environment and, surprisingly, the same respondents evaluated more positively the frequency of direct contact with the support network members. Being under full-time institutional care hampers fulfillment of psychological and social needs. Deteriorated relationships and weakened social bonds are characteristic of elderly people and are associated with their placement in care institutions. Hampered contact with family or neighbours, frequently due to great distances between the care-giving institution and the family home of an elderly person, is not conducive to building of relationships and intensifies a sense of loneliness. The research of Golden and colleagues revealed that over 1/3 of the examined respondents living in the home environment did not have any sources of support, and their inclusion in the full-time institutional care only aggravated this condition [30]. On the other hand, some lonely senior citizens, residence in a care-giving institution provided an opportunity to establish new relationships—perhaps new and unfamiliar people seemed more interesting to senior citizens and contact with them could give more pleasure. Furthermore, the fewer natural support sources in older age (of parents, partner, friends) is a natural phenomenon and one of the characteristic features of old age. Moreover, family members’ awareness of a difficult situation of an elderly person could encourage them to more intense emotional involvement and increased frequency of direct contact, and thus contribute to nurturing of family relationships. The respondents who were more often visited by family members felt needed, expressed more optimism, and coped more easily with depression caused by a change of the environment.

In the research of other authors [31,32,33] on the correlations between support and a sense of loneliness in a group of senior citizens under full-time institutional care it was demonstrated that over a half of the respondents experienced adverse consequences of loneliness and loss. These feelings were often further aggravated by death of the partner, being in mourning and a necessity to leave one’s home, where the decision about moving did not always depend on the elderly person’s will [34,35,36]. Moreover, isolation of the respondents from the local community can result from the location of a care institution. Distance between a care-giving institution and the local community of a given town or village can form a barrier to interpersonal contact. Another paradox appears here—there is noticeable isolation but at the same time a strong need to establish relationships with other people [37]. The surveys by Toczyńska and colleagues [36] confirmed that the respondents were afraid not only of loneliness but also of disability leading to lack of autonomy and to total dependence on help from other people. Moreover, the respondents’ fears pertained also to deterioration of relationships with family members in a situation of serious disease leading to less frequent visits or even to their complete cessation, by close relatives, or to lack of acceptance on their part. The level of structural support was also relatively high in these respondents, indicating a much more developed support network in comparison to deteriorated support networks of senior citizens under institutional care. According to Grzegorczyk [37] and Rodriguez and colleagues [38] depriving an elderly person of an opportunity to maintain natural bonds and support sources and replacing them with formal relationships of an institutional character, and a different lifestyle in a community, can result in the person’s gradual withdrawal from social life, shutting oneself away and experiencing emotional disturbances.

### 4.3. Demographic Determinants of Social Support

In this study, the older respondents’ (≥75 years old) assessment of structural support was worse, which can be associated with the natural phenomenon of a decline in support at old age. Kurowska and colleagues [5] and Bonior and Łysy [6] obtained similar results—younger respondents gave structural support a better assessment. This latter group can be characterised by greater independence and resourcefulness and simultaneously having little need for help from other people. Melchiorre and colleagues [7] and Winningham and Pike [8] proved that the age of ≥ 80 years old and being male are factors that require support at a higher level. Men appreciate independence and self-confidence more than women, which can hinder their acceptance of support. Male stereotypes contribute to men’s avoidance of talking about their problems and they find asking for help difficult [9]. However, men have more permanent support networks than women and they more frequently continue their relationships in networks from their youth, even if they change their living environment [10]. On the one hand, women are assumed to expect more support. On the other hand, they are givers of support that is adequate to needs. According to Litwin [39] and Haylen [40], women can utilize support given in a better way and more frequently they seem to get support from many different sources simultaneously. The results presented by Bonior and Łysy [6] correspond with our own results where women had a higher level of social interaction than men, irrespective of the place of residence.

One of the obstacles to maintaining relationships in a particular support network can be the distance between people. In old age, moving to a different place is frequently seen as a difficult or even critical situation. In this study, for the majority of the respondents moving from their own house/flat to an institution did not involve changing their town. Therefore, the individuals did not suffer much while being separated from their families, acquaintances, neighbours and so they coped with the change more easily. Additionally, seniors had a chance of establishing new relationships with new residents of care-giving institutions, which is confirmed in the research by Murphy and colleagues [41], Rash [42] and Tsai and colleagues [43]. In the aforementioned studies, respondents indicated factors that build social potential in the relationships with others and they are as follows: domestic atmosphere, integration with the local community and the possibility of being visited by family members or friends. Moreover, meeting family can be a source of emotional stability for seniors. Respondents’ satisfaction with frequent contacts with their relatives reduced their unwillingness to build relationships with other residents of care-giving institutions and encouraged them to be more engaged in the social life of the institutions. Besides, the respondents who were more frequently visited by their relatives and friends felt more important, showed more optimism, and coped with sadness caused by change of their environment more easily [44].

The results obtained by Melchiorre and colleagues [7] indicate that people who are in relationships rate the level of social contacts higher than single individuals. A partner constitutes support in difficult situations and is usually the main provider of help. On the contrary, anxiety about changes, and adaptation to them, is intensified with age. Therefore, seniors may continue their relationships all through their lives, even though they are unsatisfactory or toxic. Friends and neighbours often provide greater social support than their intimate partners [44]. The opposite tendency was observed in the authors’ own research—divorced and widowed respondents rated structural support and its components highest. In this case, divorce or widowhood can be treated as circumstances that can free them from unsatisfactory relationships. What is more, newly met and unfamiliar people may seem to be more interesting and keeping in touch with them can be more pleasurable, even if they are staying in institutions. Due to their freshness and simplicity, such relationships may seem to offer a stronger sense of participation in social life than a partner can provide. In this study, the respondents with higher education were characterised by better support in terms of structure and function, which was also confirmed by Bonior and Łysy [6].

Higher education is accompanied by higher professional and material status. Additionally, better perception of social support is attributed to individuals with higher education. It can result from better communication and interpersonal skills associated with higher education. These skills enable a more effective utilization of the support provided, and intensify the ability to establish relationships. Financial capital acquired during active professional life provides the sense of security and economic stability, which enables seniors to strengthen their relationships. Moreover, professional life facilitates building new relationships, which was also observed by Lou [45].

Support for elderly people is more effective when it takes place with involvement of small structures close to the individual. Family members’ care of a senior citizen produces much better effects than institutional care. Multidimensional support directed at elderly people turns out to be necessary in order to face challenges of protective, emotional, or informational character or in the form of support in everyday functioning.

## 5. Conclusions

1. The living environment affects the perception of social support among elderly people.
2. Full-time institutional care of a senior citizen leads to the deterioration of social support.
3. Demographic variables modify the level of seniors’ social support particularly as to structure.
4. Keeping an elderly person in the home environment as long as possible should be a priority for organization of institutional and community support.

## Figures and Tables

**Table 1:** Demographic variables in the studied groups.

Demographic Variable		Group A (*n* = 190)		Group B (*n* = 174)	
		*n*	%	*n*	%
Sex	female	99	52.11	120	68.97
	male	91	47.89	54	31.03
Age	60–74	107	56.32	90	51.72
	≥75	83	43.68	84	48.28
Education	primary	119	62.63	48	27.59
	vocational	43	22.63	55	31.61
	secondary	24	12.63	49	28.16
	higher	4	2.11	22	12.64
Marital status	single	47	24.73	32	18.39
	married	23	12.11	78	44.83
	divorced	35	18.42	0	0.00
	widowed	85	44.74	64	36.78

**Table 2:** Comparison of the results of structural support.

Quantitative Variable	Group A (*n* = 190)		Group B (*n* = 174)		Student’s *t*-Test		95% CI		*d*
	M	SD	M	SD	*t*	*p*	LL	UL	
SS	38.22	22.46	66.13	10.71	−15.33	<0.001	−31.49	−24.32	−1.59
NS	3.54	1.42	4.83	1.30	−9.02	<0.001	−1.57	−1.01	−0.95
EB	1.90	0.50	1.50	0.49	8.34	<0.001	0.31	0.49	0.81
FC	3.50	0.91	2.15	0.46	17.94	<0.001	1.19	1.49	1.87
HR	3.07	1.00	2.11	0.56	11.47	<0.001	0.80	1.13	1.18

SS—structural support, NS—network structure, EB—emotional bonds, FC—frequency of direct contact, HR—help received, M—mean, SD—standard deviation, *t*—Student’s *t*-test, *d*—Cohen’s *d* effect size, LL—lower limit, UL—upper limit.

**Table 3:** Comparison of the results of functional support.

Quantitative Variable	Group A (*n* = 190)		Group B (*n* = 174)		Student’s *t*-Test		95% CI		*d*
	M	SD	M	SD	*t*	*p*	LL	UL	
FS	48.76	12.77	44.24	11.71	3.51	0.001	1.99	7.06	0.37
INF	11.46	3.54	11.33	3.23	0.36	0.716	−0.57	0.83	0.04
INS	13.53	4.10	12.04	4.38	3.34	0.001	0.61	2.36	0.35
EVA	14.02	3.90	12.20	3.83	4.48	0.001	1.02	2.61	0.47
EMO	9.76	3.83	8.67	3.70	2.76	0.006	0.31	1.87	0.29

FS—functional support, INF—informational support, INS—instrumental support, EVA—evaluative support, EMO—emotional support, M—mean, SD—standard deviation, *t*—Student’s *t*-test, *d*—Cohen’s *d* effect size, LL—lower limit, UL—upper limit.

**Table 4:** Intergroup analysis of structural support in the context of demographic variables.

Demographic Variable	SS	NS	EB	FC	HR
Sex	*t* = 3.00	*t* = 1.93	*t* = −5.07	*t* = −3.78	*t* = −2.90
	*p* = 0.003	*p* = 0.055	*p* < 0.001	*p* < 0.001	*p* = 0.004
	*d* = 0.32	*d* = 0.21	*d* = 0.53	*d* = 0.41	*d* = 0.32
Age	*t* = −0.42	*t* = 1.68	*t* = 3.41	*t* = −0.12	*t* = −0.02
	*p* = 0.677	*p* = 0.094	*p* < 0.001	*p* = 0.903	*p* = 0.981
	*d* = 0.04	*d* = 0.16	*d* = 0.37	*d* = 0.02	*d* = 0.00
Education	H = 28.16	H = 33.81	H = 8.63	H = 30.70	H = 19.38
	*p* < 0.001	*p* < 0.001	*p* = 0.035	*p* < 0.001	*p* < 0.001
	η^2^ = 0.27	η^2^ = 0.28	η^2^ = 0.15	η^2^ = 0.27	η^2^ = 0.22
Marital status	H = 20.77	H = 202.95	H = 25.60	H = 43.50	H = 36.25
	*p* < 0.001	*p* < 0.001	*p* < 0.001	*p* < 0.001	*p* < 0.001
	η^2^ = 0.26	η^2^ = 0.72	η^2^ = 0.26	η^2^ = 0.34	η^2^ = 0.32

SS—structural support, NS—network structure, EB—emotional bonds, FC—frequency of direct contact, HR—help received, *t*—Student’s *t*-test, H—Kruskal-Wallis test, *d*—Cohen’s *d* effect size, η^2^—eta-squared effect size.

**Table 5:** Intergroup analysis of functional support in the context of demographic variables.

Demographic Variable	FS	INF	INS	EVA	EMO
Sex	*t* = −2.55	*t* = −0.85	*t* = −1.64	*t* = −2.96	*t* = −2.57
	*p* = 0.011	*p* = 0.398	*p* = 0.102	*p* = 0.003	*p* = 0.011
	*d* = 0.27	*d* = 0.09	*d* = 0.17	*d* = 0.32	*d* = 0.28
Age	*t* = 0.40	*t* = −1.04	*t* = 0.23	*t* = 0.29	*t* = 1.75
	*p* = 0.689	*p* = 0.300	*p* = 0.821	*p* = 0.775	*p* = 0.089
	*d* = 0.04	*d* = 0.11	*d* = 0.02	*d* = 0.03	*d* = 0.18
Education	H = 4.21	H = 3.77	H = 6.94	H = 5.93	H = 0.63
	*p* = 0.239	*p* = 0.287	*p* = 0.074	*p* = 0.115	*p* = 0.888
	η^2^ = 0.10	η^2^ = 0.12	η^2^ = 0.13	η^2^ = 0.12	η^2^ = 0.09
Marital status	H = 3.98	H = 2.74	H = 2.55	H = 6.22	H = 8.68
	*p* = 0.263	*p* = 0.432	*p* = 0.466	*p* = 0.101	*p* = 0.034
	η^2^ = 0.13	η^2^ = 0.09	η^2^ = 0.08	η^2^ = 0.13	η^2^ = 0.16

FS—functional support, INF—informational support, INS—instrumental support, EVA—evaluative support, EMO—emotional support, *t*—Student’s *t*-test, H—Kruskal-Wallis test, *d*—Cohen’s d effect size, η^2^—eta-squared effect size.
